# Glycerol Monolaurate Ameliorated Intestinal Barrier and Immunity in Broilers by Regulating Intestinal Inflammation, Antioxidant Balance, and Intestinal Microbiota

**DOI:** 10.3389/fimmu.2021.713485

**Published:** 2021-09-23

**Authors:** Linglian Kong, Zhenhua Wang, Chuanpi Xiao, Qidong Zhu, Zhigang Song

**Affiliations:** ^1^ Department of Animal Science and Technology, Shandong Agricultural University, Taian, China; ^2^ Center for Mitochondria and Healthy Ageing, College of Life Sciences, Yantai University, Yantai, China; ^3^ Precision Livestock and Nutrition Unit, Gembloux Agro-Bio Tech, University of Liège, Gembloux, Belgium

**Keywords:** glycerol monolaurate, intestinal barrier, antioxidant, inflammation, microbiota

## Abstract

This study was conducted to investigate the impact of glycerol monolaurate (GML) on performance, immunity, intestinal barrier, and cecal microbiota in broiler chicks. A total of 360 one-day-old broilers (Arbor Acres) with an average weight of 45.7 g were randomly allocated to five dietary groups as follows: basal diet and basal diets complemented with 300, 600, 900, or 1200 mg/kg GML. Samples were collected at 7 and 14 days of age. Results revealed that feed intake increased (*P* < 0.05) after 900 and 1200 mg/kg GML were administered during the entire 14-day experiment period. Dietary GML decreased (*P* < 0.05) crypt depth and increased the villus height-to-crypt depth ratio of the jejunum. In the serum and jejunum, supplementation with more than 600 mg/kg GML reduced (*P* < 0.05) interleukin-1β, tumor necrosis factor-α, and malondialdehyde levels and increased (*P* < 0.05) the levels of immunoglobulin G, jejunal mucin 2, total antioxidant capacity, and total superoxide dismutase. GML down-regulate (*P* < 0.05) jejunal interleukin-1β and interferon-*γ* expression and increased (*P* < 0.05) the mRNA level of zonula occludens 1 and occludin. A reduced (*P* < 0.05) expression of toll-like receptor 4 and nuclear factor kappa-B was shown in GML-treated groups. In addition, GML modulated the composition of the cecal microbiota of the broilers, improved (*P* < 0.05) microbial diversity, and increased (*P* < 0.05) the abundance of butyrate-producing bacteria. Spearman’s correlation analysis revealed that the genera *Barnesiella*, *Coprobacter*, *Lachnospiraceae*, *Faecalibacterium*, *Bacteroides*, *Odoriacter*, and *Parabacteroides* were related to inflammation and intestinal integrity. In conclusion, GML ameliorated intestinal morphology and barrier function in broiler chicks probably by regulating intestinal immune and antioxidant balance, as well as intestinal microbiota.

## Introduction

Antibiotics play a significant role in disease prevention and growth promotion in the poultry industry. Despite increasing demand for poultry, antibiotic residues and resistance issues led to the ban of antibiotic growth promoters (1), pressuring the industry to find alternatives for maintaining poultry flock health. One promising approach is immune modulation, in which natural host mechanisms are exploited to enhance and modulate bird’s immune response (2). Targeted dietary supplementation or using a feed additive may be useful in the immunomodulation of the immune system. These ingredients can reduce the negative impacts of environmental stressors on animal immune systems and production performance (3). For instance, antimicrobial peptides are feed additives that neutralize lipopolysaccharide (LPS) from *Pseudomonas aeruginosa* at the cellular level and significantly inhibit tumor necrosis factor (TNF-α) and nitric oxide production in the macrophages of LPS-treated mice (4). Extensive research has been carried out to evaluate an array of products as alternatives to antibiotic growth promoters; such products, including food industry by-products, plant metabolites, non-digestible oligosaccharides, natural by-products, essential minerals, amino acids, medicinal herbs, organic acids, and essential oils, can at least partially alter immune function in poultry (2).

Glycerol monolaurate (GML), a fatty acid composed of glycerol and lauric acid, possesses a large range of immunoregulatory properties (5). GML is considered a food-safe emulsifier endorsed by the Food and Drug Administration and recognized as a nontoxic compound even at relatively high dose levels (6). GML possesses potent antibacterial properties with inhibitory activity against the production of MIP-3α and secretion of proinflammatory cytokines (7). *In vitro*, GML suppresses T cell receptor (TCR)-induced signaling and T cell effector function to reduce the production of cytokines induced by TCR (5). Moreover, GML can inhibit mucosal signaling and the innate and inflammatory response to human immunodeficiency virus 1, which indicated the immunoregulatory effect of GML (8). Recent studies revealed that GML is an efficacious antibiotic growth promoter alternative for animal health and has the potential to become a unique fungicide owing to its established safety, antibacterial properties, and immunomodulatory capacity (9, 10). The immune status and intestinal histomorphology of broilers can be enhanced by GML with a supplementary dosage of up to 5 g/kg to basal diets (3).

Despite the potential of GML as an additive in broiler feed to change meat quality (11) and fatty acid profile (12), the information on the influence of GML on the immune and antioxidant response of broilers is insufficient. In an oral cell/bacteria co-culture dual-chamber model, GML modulated the host immune response and metabolite production, which demonstrated that GML could be considered a potential candidate for *in vivo* studies (13). Extensive interactions occur between a poultry host and its gut microbiome, particularly during exchange of nutrients and modulation of host gut morphology, physiology, and immunity (14). Growth performance, muscle amino acids, and intestinal development are improved by dietary GML in mice and laying hens through intestinal microbiota regulation (6, 15, 16). Thus, the hypothesis of the present study was that GML can improve the immunity and intestinal barrier of broilers by altering the gut microbiota. The objective of this study was to evaluate the effects of different dosages of GML on performance, immunity, intestinal barrier, antioxidant capacity, and microbiota in broilers.

## Materials and Methods

### Animals, Experimental Design and Management

A total of 360 one-day-old broilers (Arbor Acres) with an average weight of 45.7 g were randomly divided into five groups as follows: basal diet (control) and basal diets supplemented with 300, 600, 900, or 1200 mg/kg GML, which was purchased from Henan Zhengtong Food Technology Co., Ltd (Henan, China) with purity of more than 90%. The additive dosage of GML was optimized according to previous studies (3, 6, 17). Each group contained six replicates (cage) of 12 broilers per cage. The ingredients and nutrients levels in the basal diet were formulated according to standards of the National Research Council 2012 (**Table 1**). All broilers were weighed and randomly assigned to 30 metal cages (70 cm × 70 cm × 40 cm), which were equipped with feeders and nipple drinkers. Broilers with similar initial weights were reared in an environmentally controlled room. The temperature was 35°C initially, and then gradually decreased to 28°C until the end of the 14-day experiment period. In the first 3 days, average relative humidity was maintained at approximately 70%, and thereafter maintained between 55% and 65%. In the first week, the broilers were kept under 23 h of light and 1 h of darkness, which were then gradually reduced to 20 and 4 h, respectively.

**Table 1:** Ingredient composition and nutritional components of the basal diet.

Items	Content (%)
Ingredient	
Corn	60.00
Soybean meal (43)	28.80
Corn protein flour (60)	5.30
Salt	0.16
Baking soda	0.20
Limestone	1.30
Dicalcium phosphate	0.75
Soybean oil	2.20
Vitamin premix	0.03
Mineral premix	0.20
Choline chloride (50%)	0.10
Methionine	0.23
Lysine (70%)	0.58
Threonine (98.5%)	0.134
Phytase (20000U)	0.02
Total	100.00
Nutritional level	
Metabolizable energy	2850.00 (kcal/kg)
Crude protein	21.50
Lysine	1.26
Methionine	0.57
Calcium	0.60
Total phosphorus	0.60
Available phosphorus	0.40

Provided for per kilogram of compound diet: vitamin A, 12000 IU; vitamin D3, 5000 IU; vitamin E, 80 mg; VK, 3.2 mg; vitamin B1, 3.2 mg; vitamin B2, 8.6 mg; nicotinic acid, 65 mg; pantothenic acid, 20 mg; vitamin B6, 4.3 mg; biotin, 0.22 mg; folic acid, 2.2 mg; vitamin B12, 0.017 mg; I, 1.25 mg; Fe, 20 mg; Mn, 120 mg; Se, 0.3 mg; Zn, 110 mg. Nutrition level was the calculated value.

### Growth Performance

On days 7 and 14, feed consumption in each replicate and body weight were recorded. Body weight gain (BWG) and feed intake (FI) were calculated subsequently. Spilled feed was carefully collected and weighed for the correction of the final FI data. Feed conversion rate (FCR) was defined as FI : BWG. The data of mortality were recorded and included in the FCR calculation.

### Sampling

Two broilers per replicate were randomly selected for sampling after growth performance was determined on 7 and 14 days of age. Blood samples were collected from wing veins and negotiated to glass tubes without anticoagulants, then centrifuged at 3000 rpm for 10 min at 4°C. Serum was obtained and stored at −20°C for biochemical analysis. Broilers were slaughtered by cervical dislocation after blood samples were obtained. Approximately 2 cm segments were excised from the jejunum (from the entry point of the bile duct to the Meckel’s diverticulum), flushed repeatedly with cold saline solution, and immediately immersed in 4% paraformaldehyde solution for histological examination. Tissue samples (1–2 g) were collected from the jejunum, rapidly frozen in liquid nitrogen, and stored at −80°C for molecular analysis. The cecum was collected on ice, frozen quickly in liquid nitrogen, transported to the laboratory in a dry-ice bag, and then stored at −80°C for further microbial analysis.

### Jejunal Morphology Analysis

Jejunum segments were fixed in 4% paraformaldehyde solution for 24 h, dehydrated, and embedded in paraffin. Tissue sections with 5 μm thickness were cut using a microtome (Leica RM2235, Leica Biosystems Inc., Buffalo Grove, USA), fixed on slides, and stained with hematoxylin and eosin. The images of the jejunum were analyzed with ImageJ analysis software (Version 1.47, Bethesda, MD, USA). Ten intact villi were selected randomly from each section for morphology measurement. Villus height (VH) was gauged from the tip of the villus to the villus–crypt junction. Crypt depth (CD) was defined as the depth of the invagination between adjacent villi. Villus height-to-crypt depth ratio (VCR) was calculated. The mean value of ten values attributed to individual broilers was used in statistical analysis.

### Biochemical Assay of Serum and Jejunum

Immune response status in the sera was estimated by detecting the levels of interleukin 1 beta (IL-1β), interleukin 6 (IL-6), tumor necrosis factor-alpha (TNF-α), and immunoglobulin G (IgG) with ELISA kits (MLBIO Co., Ltd., Shanghai, China). All determination procedures were performed strictly according to the manufacturer’s instructions. The inter- and intra-assay coefficients of variation (CVs) were less than 10%. The jejunal samples were weighed accurately (0.3 g), homogenized with 2.7 ml phosphate-buffered saline in a weight (g): volume (ml) ratio of 1: 9. The homogenates were centrifuged at 1000 g for 10 min at 4°C, and the supernatants were collected for the detection of secreted immunoglobulin A (sIgA) and mucin 2 (MUC 2) levels with ELISA kits (MLBIO Co., Ltd., Shanghai, China). The results were expressed as pg/mg of protein.

### Antioxidant Assay of Serum and Jejunum

Malondialdehyde (MDA) levels, total antioxidant capacity (T-AOC), and total superoxide dismutase (T-SOD) activity were measured in each serum and jejunal homogenate with diagnostic kits (intra-assay CV < 5%; inter-assay CV < 8%) purchased from Nanjing Jiancheng Biotechnology Institute (Nanjing, China) according to the manufacturer’s instructions. The results were normalized to protein concentration in each jejunal homogenate.

### RNA Isolation and Quantitative Polymerase Chain Reaction (qPCR)

The total RNA of the jejunum was isolated using Trizol reagent (Invitrogen Biotechnology Inc., CA, USA). RNA quality was evaluated through 1% agarose gel electrophoresis. The reverse transcription of 1 μg of total RNA was performed using PrimeScript^®^ RT reagent kit with gDNA Eraser (RR047A, Takara Bio Inc., Dalian, China). The gene expression was determined through RT-PCR. TB Green Premix Ex Taq (RR820A, Takara Bio Inc., Dalian, China) and ABI 7500 real-time PCR systems (Applied Biosystems, CA, USA) were used. The reaction program was as follows: predenaturation at 95°C for 10 s, then denaturation at 95°C for 5 s for a total of 40 cycles, and finally annealing and extension at 60°C for 34 s. Each reaction was repeated three times, and the primer sequences are shown in **Table 2**. The amplification efficiency of the primers was calculated with a standard curve. The specificity of the amplified products was verified with the melting curve. The relative expression of the target gene was analyzed through the 2^−ΔΔCt^ method after normalization against the geometric mean of the expression of β-actin, glyceraldehyde-3-phosphate dehydrogenase (GAPDH), and TATA-binding protein (TBP).

**Table 2:** Nucleotide sequences for real-time PCR primers.

Gene	Accession Number	Primer sequence, 5′→ 3′	Product size (bp)
*IL-1β*	NM_204524.1	GGTCAACATCGCCACCTACA	86
		CATACGAGATGCAAACCAGCAA	
*IL-6*	NM_204628.1	CCTTTCAGACCTACCTGGAATT	130
		ACTTCATCGGGATTTATCACCA	
*TNF-α*	NM_204267.1	TGTGTATGTGCAGCAACCCGTAGT	229
		GGCATTGCAATTTGGACAGAAGT	
*IFNγ*	NM_205149.1	TGAGCCAGATTGTTTCGATG	246
		TCCTTTTGAAACTCGGAGGA	
*NF-κB*	XM_015285418.2	CACGGAGGCTTGATCCTGTT	96
		CCGCTGTCCTGTCCATTCTT	
*TLR4*	NM_001030693.1	AGTCTGAAATTGCTGAGCTCAAAT	190
		GCGACGTTAAGCCATGGAAG	
*ZO-1*	XM_015278981.2	CTTCAGGTGTTTCTCTTCCTCCTCTC	131
		CTGTGGTTTCATGGCTGGATC	
*occludin*	NM_205128.1	GCTCTGCCTCATCTGCTTCTT	142
		CCCATCCGCCACGTTCTTC	
*claudin-1*	NM_001013611.2	CTGATTGCTTCCAACCAG	140
		CAGGTCAAACAGAGGTACAAG	
*claudin-2*	NM_001277622.1	CCTGCTCACCCTCATTGGAG	145
		GCTGAACTCACTCTTGGGCT	
*GAPDH*	NM_204305.1	GCCCAGAACATCATCCCA	137
		CGGCAGGTCAGGTCAACA	
*β-actin*	NM_205518.1	CACCACAGCCGAGAGAGAAA	215
		CACAGGACTCCATACCCAAGAA	
*TBP*	AF221563	AGCTCTGGGATAGTGCCACAG	134
		ATAATAACAGCAGCAAAACGCTTG	

IL-1β, interleukin 1β; IL-6, interleukin 6; TNF-α, tumor necrosis factor α; TLR4, toll-like receptor; NF-κB, nuclear factor kappa-B; IFN-γ, interferon γ ; GAPDH, glyceraldehyde-3-phosphate dehydrogenase; TBP, TATA-binding protein.

### 16S rRNA Sequencing and Analysis

Total DNA was extracted from cecal contents with an E.Z.N.A.^®^ Soil DNA kit (Omega Bio-Tek, Norcross, GA, U.S.) according to the manufacturer’s instructions. DNA purity and concentration were evaluated with a Nano Drop2000 spectrophotometer (Thermo Scientific, Wilmington, USA), and DNA integrity was detected through 1% agarose gel electrophoresis. Bacterial 16S rRNA gene spanning the V3-V4 hypervariable regions were amplified with primers 338F (5′-ACTCCTACGGGAGGCAGCAG-3′) and 806R (5′-GGACTACHVGGGTWTCTAAT-3′) with a PCR system. PCR reactions were performed in triplicate with a 20 μl mixture consisting of 4 μl of 5 × FastPfu Buffer, 2 μl of 2.5 mM dNTPs, 0.8 μl of each primer (5 μM), 0.4 μl of FastPfu polymerase, and 10 ng of template DNA. The amplification programs were set in ABI GeneAmp^®^ 9700 system (ABI, USA) as follows: 3 min at 95°C, 27 cycles of 30 s at 95°C, 55°C for 30 s, and 72°C for 45 s, and 72°C for 10 min. The PCR products were detected through 2% agarose gel electrophoresis and purified with an AxyPrep DNA gel recovery kit (Axygen Biosciences, Union City, CA, USA) and then quantified with a QuantiFluor™-ST blue fluorescence quantitative system (Promega, USA). Purified amplicons were pooled in equimolar amounts, and their paired-end reads were sequenced on an Illumina MiSeq PE300 platform (Illumina, San Diego, USA) by Majorbio Bio-Pharm Technology Co., Ltd. (Shanghai, China). Raw fastq files obtained through MiSeq sequencing were demultiplexed, quality-filtered, trimmed, de-noised with trimmomatic, and merged ​​according to the overlapping relationship by FLASH (version 1.2.11, https://ccb.jhu.edu/software/FLASH/index.shtml). The filtered reads were clustered into operational taxonomic units (OTUs) with a 97% sequence identity with UPARSE (version 7.1, http://www.drive5.com/uparse/). Chimera was removed during clustering. The OTU representative sequence was analyzed with the RDP Classifier (version 2.2, http://sourceforge.net/projects/rdp-classifier/) against the Silva 16S rRNA database (release119, http://www.arb-silva.de) at a confidence threshold of 70%.

### Statistical Analysis

All data were checked for normality with Shapiro–Wilk test (95% confidence level) and subjected to ANOVA using the GLM procedure of SPSS software (SPSS 26.0, SPSS, Chicago, USA). Orthogonal polynomial contrasts were performed to determine linear and quadratic responses to the increasing level of dietary GML. Statistical differences between groups were analyzed with Tukey’s multiple comparisons followed by unpaired Wilcoxon comparison test, as well as by the non-parametric factorial Kruskal-Wallis test. Differences were considered significantly different at *P* < 0.05.

## Results

### Effects of Dietary GML on Growth Performance

To evaluate the effects of dietary GML supplementation on the growth performance of broilers, the FI, BW, BWG, and FCR were recorded and calculated at different phases (**Table 3**). After 7–14 days of treatment, the FI linearly increased in the 600, 900, and 1200 mg/kg GML-treated groups compared with those in the 300 mg/kg group (*P* < 0.05) but showed no significant difference from those of the control group (*P* > 0.05). The administration of 900 and 1200 mg/kg GML increased FI compared to the control and 300 mg/kg GML-treated group during the overall period (linear, *P* = 0.014). Dietary GML did not affect the BW, BWG, and FCR of broiler chicks (*P* > 0.05).

**Table 3:** Effects of dietary treatment on growth performance in 7- and 14-day-old broilers.

Items	GML levels (mg/kg feed)					SEM	*P*-value	
	CON	300	600	900	1200		Linear	Quadratic
BW (g/bird)								
1d	45.71	45.65	45.66	45.67	45.67	0.04	0.795	0.748
7d	154.82	151.19	148.71	150.00	152.99	1.09	0.535	0.075
14d	401.42	389.23	392.67	392.54	397.07	1.71	0.892	0.149
FI (g/bird)								
1 to 7d	145.74	148.60	143.01	150.34	155.88	1.17	0.937	0.270
7 to 14d	354.74^ab^	337.05^b^	367.62^a^	375.46^a^	366.22^a^	3.79	0.018	0.988
1 to 14d	500.04^bc^	485.65^c^	511.82^b^	525.64^a^	521.54^a^	3.79	0.014	0.794
BWG (g/bird)								
1 to 7d	116.11	120.54	118.05	119.33	122.32	1.02	0.348	0.083
7 to 14d	237.60	221.38	228.95	227.54	229.08	2.08	0.384	0.138
1 to 14d	358.32	343.31	347.00	346.88	351.40	2.46	0.492	0.126
FCR (g/g)								
1 to 7d	1.24	1.22	1.21	1.29	1.19	0.01	0.661	0.404
7 to 14d	1.49	1.56	1.60	1.60	1.56	0.02	0.189	0.11
1 to 14d	1.42	1.44	1.49	1.51	1.46	0.01	0.153	0.104

BW, body weight; FI, feed intake; FCR, feed conversion ratio; SEM, total standard error of mean.

Values were expressed as least squares means ± SEM (n = 6).

^a,b,c^Different superscripts in the same line indicate significant differences according to Tukey’s multiple comparisons (P < 0.05).

### Dietary GML Affected Cytokine Production and IgG levels in the Serum

Immunomodulatory effects have been discovered in glycerol-esters of MCFAs (17). To initially evaluate the effect of dietary GML on immune response status, the levels of IL-1β, IL-6, TNF-α, and IgG in the serum were estimated by ELISA (**Table 4**). On day 7, dietary GML linearly reduced serum IL-1β and TNF-α levels (*P* < 0.05). Significant differences in these two types of pro-inflammatory cytokine were observed in the 1200 mg/kg GML-treated group compared with the control group (*P* < 0.05). On day 14, dietary treatment with 600, 900, and 1200 mg/kg GML linearly and quadratically reduced serum IL-1β level compared with the level in the control group (*P* < 0.05). In addition, 1200 mg/kg GML increased serum IgG levels relative to those of the control (linear, *P* = 0.002). The immunomodulatory capacity of GML was confirmed as evidenced by alleviated inflammation and improved immunoglobulin levels.

**Table 4:** Effects of dietary treatment on immune indicators in the sera of 7- and 14-day-old broilers.

Items	GML levels (mg/kg feed)					SEM	*P*-value	
	CON	300	600	900	1200		Linear	Quadratic
7 d of age (pg/mL)								
IL-1β	597.46^a^	559.36^ab^	531.91^ab^	541.96^ab^	525.78^b^	8.42	0.004	0.197
IL-6	29.05	28.90	28.62	29.73	29.57	0.36	0.530	0.693
TNF-α	81.37^a^	79.03^ab^	78.27^ab^	75.44^ab^	72.75^b^	0.92	0.001	0.707
IgG	1934.38	1911.25	1832.50	1953.13	1829.38	24.29	0.331	0.993
14 d of age (pg/mL)								
IL-1β	641.47^a^	590.74^ab^	566.23^b^	531.36^b^	560.10^b^	9.25	<0.001	0.014
IL-6	29.49	29.63	29.63	30.96	30.20	0.37	0.336	0.891
TNF-α	76.90	73.69	78.36	77.96	78.21	0.84	0.254	0.792
IgG	1832.13^b^	1813.13^b^	1941.88^ab^	1939.38^ab^	2115.00^a^	32.09	0.002	0.315

IL-1β, interleukin 1β; IL-6, interleukin 6; TNF-α, tumor necrosis factor α; IgG, immunoglobulin G; SEM, total standard error of mean.

Values were expressed as least squares means ± SEM (n = 12).

^a,b^Different superscripts in the same line indicate significant differences according to Tukey’s multiple comparisons (P < 0.05).

### Dietary GML Led to Reduced Crypt Depth and Augmented MUC 2 Levels

Intestinal morphology parameters play a significant role in nutrient digestion and absorption, which are defined as important indicators of intestinal health, recovery, and function (18). Thus, maintaining intestinal barrier function might be key in the GML-protected effects on intestinal health. The intestinal morphology in the jejunum was measured to investigate the effect of GML on intestinal integrity (**Table 5**). Hematoxylin and eosin staining revealed that GML linearly decreased CD and increased VCR in the jejunum on days 7 and 14 with increasing dosages (*P* < 0.05). Moreover, the levels of sIgA and MUC 2 in the jejunum were examined. Diet complemented with 1200 mg/kg GML linearly and quadratically increased jejunal MUC 2 levels relative to those of the control on day 14 (*P* < 0.05). No alterations in jejunal VH and sIgA were observed after dietary treatment with GML (*P* > 0.05). These findings suggested that GML can promote intestinal morphology and barrier function of broiler chicks.

**Table 5:** Effects of dietary treatment on the jejunal morphology, sIgA, and MUC 2 of 7- and 14-day-old broilers.

Items	GML levels (mg/kg feed)					SEM	*P*-value	
	CON	300	600	900	1200		Linear	Quadratic
7 d of age								
VH (µm)	659.86	708.71	706.49	684.76	675.15	14.75	0.881	0.300
CD (µm)	107.01^a^	101.01^a^	83.39^ab^	67.32^b^	60.48^b^	4.37	<0.001	0.928
VCR	6.24^d^	7.11^cd^	8.66^bc^	10.34^ab^	11.30^a^	0.43	<0.001	0.841
sIgA (ng/mgprot)	26.01	26.48	25.70	26.60	25.32	0.45	0.694	0.645
MUC 2 (ng/mgprot)	209.22	208.11	201.65	202.80	198.44	2.28	0.14	0.97
14 d of age								
VH (µm)	745.08	697.30	670.03	686.95	708.35	15.99	0.663	0.227
CD (µm)	102.33^a^	89.90^ab^	73.28^bc^	66.33^c^	69.10^c^	3.44	<0.001	0.019
VCR	7.30^b^	7.84^ab^	8.88^ab^	10.37^a^	10.34^a^	0.39	0.001	0.764
sIgA (ng/mgprot)	25.18	25.10	27.62	25.35	25.73	0.38	0.611	0.221
MUC 2 (ng/mgprot)	194.11^b^	192.53^b^	202.68^ab^	201.31^ab^	212.2^a^	2.14	0.002	0.418

VH, villus height; CD, crypt depth; VCR, villus height to crypt depth ratio; sIgA, secreted immunoglobulin A; MUC 2, mucin 2; mgprot, mg of protein; SEM, total standard error of mean.

Values were expressed as least squares means ± SEM (n = 6).

^a,b,c,d^Different superscripts in the same line indicate significant differences according to Tukey’s multiple comparisons (P < 0.05).

### Intestinal Gene Expression

To further investigate the genetic basis of the effect of GML on intestinal barrier, the expression of genes related to cytokines, innate immune, and tight junction was determined in the jejunum by q-PCR (**Table 6**). On 7-day-old broilers, dietary treatment with 1200 mg/kg GML down-regulated jejunal *IL-1β* (linear, *P* = 0.008) and *interferon(IFN-γ)* (linear, *P* = 0.002; quadratic, *P* = 0.021) expression compared with that in the control. Moreover, 600 mg/kg GML-treated broilers showed higher *zonula occludens (ZO)-1* expression levels than the control (quadratic, *P* = 0.002). The mRNA level of *occludin* quadratically increased in the jejunum with 300, 600, and 1200 mg/kg GML supplementation (*P* < 0.05). On 14-day-old broilers, *IL-6* expression was lower in the 1200 mg/kg group (*P* < 0.05). The relative gene expression of *IFN-γ* was linearly and quadratically increased with increasing dietary GML levels (*P* < 0.05). Jejunal *occludin* expression was not altered by GML supplementation, compared with that in the control (*P* > 0.05), but a difference in jejunal *occludin* expression between GML-treated groups was observed (linear, *P* = 0.009; quadratic, *P* = 0.016). The mRNA expression levels of toll-like receptor4 (TLR4) were decreased in 600 and 1200 mg/kg GML-treated broilers (linear, *P* = 0.049). The translocation of nuclear factor kappa-B (NF-κB) from the cytosol into the nucleus is the critical step in many inflammatory processes (19). Dietary GML linearly reduced the expression of jejunal NF-κB in 14-day-old broilers (*P* < 0.05). These data indicate that GML-protected effects on intestinal health involved the reduction of inflammation, maintenance of intestinal barriers, and regulation of the TLR4/NF-κB signaling pathway.

**Table 6:** Effects of dietary treatment on jejunal gene expression of 7- and 14-day-old broilers.

Items	GML levels (mg/kg feed)					SEM	*P*-value	
	CON	300	600	900	1200		Linear	Quadratic
7 d of age								
*IL-1β*	1.00^a^	0.77^ab^	1.04^a^	0.80^ab^	0.55^b^	0.05	0.008	0.178
* IL-6*	1.00	1.38	1.41	1.32	1.16	0.07	0.728	0.135
* TNF-α*	1.00	1.04	0.97	0.86	0.81	0.04	0.049	0.645
* IFN-γ*	1.00^a^	0.96^ab^	1.28^a^	0.85^ab^	0.59^b^	0.08	0.091	0.021
* ZO-1*	1.00^b^	1.13^ab^	1.31^a^	1.17^ab^	1.03^ab^	0.04	0.301	0.002
* occludin*	1.00^b^	1.54^a^	1.31^a^	1.04^b^	1.31^a^	0.05	0.609	0.075
* claudin-1*	1.00	0.99	1.29	1.02	1.35	0.07	0.238	0.923
* claudin-2*	1.00	0.96	1.22	1.22	1.17	0.07	0.302	0.644
* TLR4*	1.00	1.06	0.91	0.83	1.01	0.04	0.461	0.389
* NF-κB*	1.00	0.97	1.11	1.01	0.88	0.04	0.438	0.185
14 d of age								
* IL-1β*	1.00	0.71	0.75	0.79	0.80	0.07	0.322	0.220
* IL-6*	1.00^a^	0.87^ab^	0.84^ab^	1.09^a^	0.65^b^	0.05	0.071	0.529
* TNF-α*	1.00	0.94	1.04	0.88	1.07	0.04	0.839	0.414
* IFN-γ*	1.00^a^	0.61^b^	0.52^b^	0.51^b^	0.45^b^	0.06	0.006	0.047
* ZO-1*	1.00	1.02	1.13	1.20	1.34	0.04	0.009	0.555
* occludin*	1.00^abc^	0.86^bc^	0.77^c^	1.26^a^	1.13^ab^	0.04	0.009	0.016
* claudin-1*	1.00	0.92	0.88	1.09	0.84	0.05	0.501	0.964
* claudin-2*	1.00	0.99	1.00	1.24	1.24	0.06	0.102	0.544
* TLR4*	1.00^a^	0.69^ab^	0.63^b^	0.82^ab^	0.65^b^	0.05	0.049	0.102
* NF-κB*	1.00^a^	0.70^ab^	0.79^b^	0.81^ab^	0.74^b^	0.03	0.040	0.084

IL-1β, interleukin 1β, IL-6, interleukin 6; TNF-α, tumor necrosis factor α; TLR4, toll-like receptor; NF-κB, nuclear factor kappa-B; IFN-γ, interferon γ ; GAPDH, glyceraldehyde-3-phosphate dehydrogenase; TBP, TATA-binding protein; SEM, total standard error of mean.

Values were expressed as least squares means ± SEM (n = 6).

^a,b,c^Different superscripts in the same line indicate significant differences according to Tukey’s multiple comparisons (P < 0.05).

### Effects of GML on Serum and Jejunal Antioxidant Indexes

Important connections between inflammation and oxidative stress have been demonstrated (20). Since consumption of diet containing GML led to changes in gene expression of cytokines in the jejunum, whether GML diet would also affect antioxidant indexes was next examined (**Table 7**). Dietary GML linearly inhibited the production of MDA in the serum and jejunum on 14-day-old broilers (*P* < 0.05). Serum SOD level linearly and quadratically increased in broilers fed with 1200 mg/kg GML on day 7 relative to that of the control and linearly improved 14 days after GML supplementation (*P* < 0.05). In the jejuna of 7-day-old broilers, dietary treatment with GML linearly increased SOD levels (*P* < 0.05), and the highest level was found in the 1200 mg/kg group (*P* < 0.05). A higher T-AOC level was observed in the jejuna of the 1200 mg/kg groups on day 7 (linear, *P* = 0.01) and in the sera after 900 and 1200 mg/kg GML addition on day 14 (linear, *P* = 0.002). These data revealed the antioxidant-enhancing effects of GML.

**Table 7:** Effects of dietary treatment on antioxidant capacity in 7- and 14-day-old broilers.

Items	GML levels (mg/kg feed)					SEM	*P*-value	
	CON	300	600	900	1200		Linear	Quadratic
Serum, 7 d of age								
MDA (nmol/ml)	5.88	5.29	5.99	5.67	5.08	0.19	0.353	0.506
SOD (U/ml)	67.41^b^	71.9^ab^	65.4^b^	82.87^ab^	93.84^a^	3.84	0.196	0.009
T-AOC (mmol/ml)	0.56	0.54	0.57	0.58	0.63	0.02	0.143	0.449
Serum, 14 d of age								
MDA (nmol/ml)	6.55^a^	4.64^b^	4.07^b^	4.51^b^	4.95^b^	0.22	0.012	0.001
SOD (U/ml)	75.48^b^	84.33^ab^	89.43^ab^	101.29^ab^	116.44^a^	4.31	0.001	0.534
T-AOC (mmol/ml)	0.41^b^	0.47^ab^	0.49^ab^	0.52^a^	0.52^a^	0.01	0.002	0.272
Jejunum, 7 d of age								
MDA (nmol/mgprot)	0.41	0.49	0.52	0.44	0.57	0.03	0.206	0.955
SOD (U/mgprot)	2.62^c^	3.35^bc^	3.84^ab^	3.6^ab^	4.23^a^	0.12	<0.001	0.225
T-AOC (mmol/mgprot)	0.08^b^	0.11^ab^	0.13^ab^	0.12^ab^	0.18^a^	0.01	0.010	0.898
Jejunum, 14 d of age								
MDA (nmol/mgprot)	0.85^a^	0.6^ab^	0.56^b^	0.55^b^	0.56^b^	0.03	0.004	0.025
SOD (U/mgprot)	3.72	3.77	3.76	4.01	3.50	0.11	0.855	0.399
T-AOC (mmol/mgprot)	0.09	0.08	0.08	0.08	0.07	0.01	0.335	0.678

MDA, malondialdehyde; SOD, superoxide dismutase; T-AOC, total antioxidant capacity; mgprot, mg of protein; SEM, total standard error of mean.

Values were expressed as least squares means ± SEM (n = 12).

^a,b,c^Different superscripts in the same line indicate significant differences according to Tukey’s multiple comparisons (P < 0.05).

### Composition and Community Diversity of Cecal Microbiota

**Figure 1:**
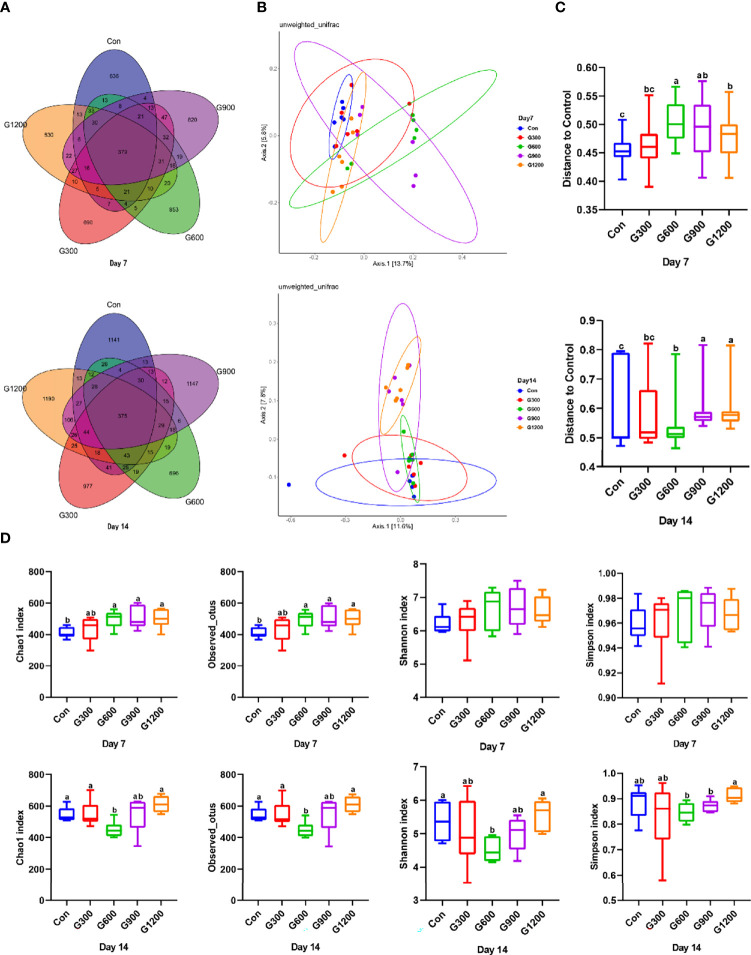
Dietary GML altered the composition and community diversity of cecal microbiota. **(A)** Venn diagram between treatments on OTUs level. **(B)** PCoA analysis based on unweighted Unifrac distance. **(C)** PERMANOVA analysis based on unweighted Unifrac distance. **(D)** Alpha-diversity based on indices of Chao 1, Observed otus, Shannon and Simpson; different superscripts indicate significant differences according to the unpaired Wilcoxon comparison test. Con, basal diet; G300, 600, 900, and 1200, basal diets complemented with 300, 600, 900, or 1200 mg/kg GML.

At the end of the study, 16S rRNA gene high-throughput sequencing was performed to reveal the impact of GML on the cecal microbiota. Generation of a Venn diagram showed that 379 OTUs were found among the five groups, and 636, 690, 853, 820, and 830 specific OTUs were unique to the control, 300, 600, 900, and 1200 mg/kg GML-treated groups on 7-day-old broilers, respectively (**Figure 1A**). On 14 days of age, 1141, 977, 696, 1147, and 1190 specific OTUs existed respectively in the Con, 300, 600, 900, and 1200 mg/kg GML-treated groups, with 375 OTUs shared (**Figure 1A**). A principal coordinate analysis (PCoA) was performed to assess similarities and differences among samples and groups (**Figure 1B**). The results indicated distinct clusters of microbiota composition among the five groups. PERMANOVA analysis based on unweighted unifrac distance was performed to quantify the differences in species diversity. The data revealed that higher than or equal to 600 mg/kg GML significantly altered the β diversity index compared to the control group (**Figure 1C**). The Chao 1 index and Observed OTUs revealed that diets supplemented with 600, 900, and 1200 mg/kg GML significantly increased the alpha diversity of 7-day-old broilers microbiota (**Figure 1D**). However, reduced Chao 1, Observed OTUs, Shannon, and Simpson indices were observed in 600 mg/kg GML-treated broilers on 14 days of age (**Figure 1D**). These results indicated that dietary GML modulated the cecal microbiota community structure of broiler chicks.

**Figure 2:**
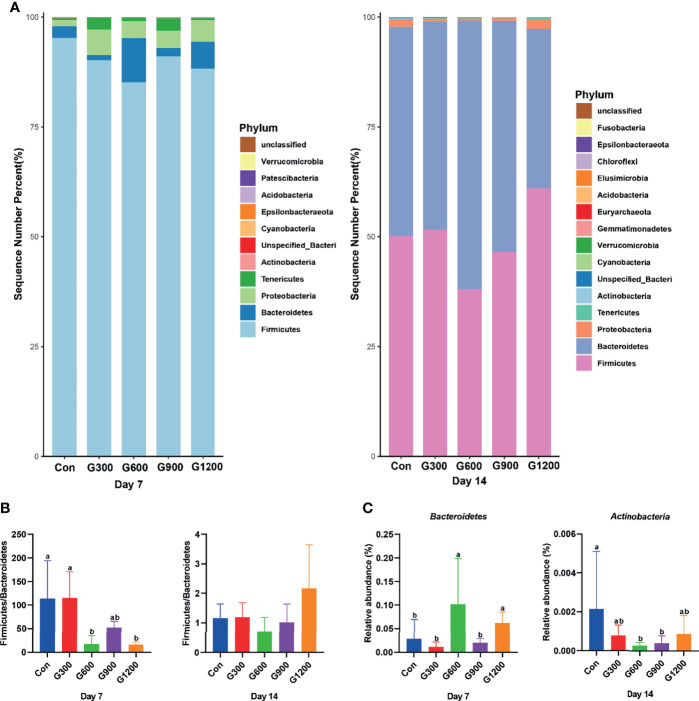
Alteration of cecal microbiota on the phylum level. **(A)** The top 20 phyla in the relative abundance of each group. **(B)** The ratio of Firmicutes/Bacteroidetes. **(C)** Relative abundance of *Bacteroidetes* and *Actinobacteria*. Different superscripts indicate significant differences according to the Kruskal-Wallis test (*P* < 0.05). Con, basal diet; G300, 600, 900, and 1200, basal diets complemented with 300, 600, 900, or 1200 mg/kg GML.

**Figure 3:**
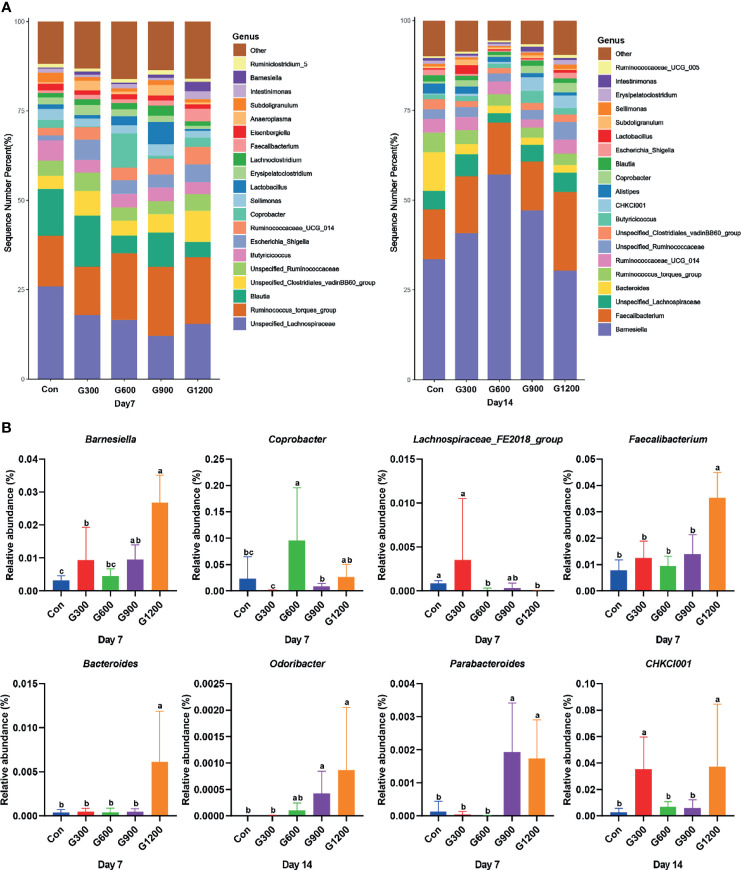
Alteration of cecal microbiota on the genus level. **(A)** Relative abundance of top 20 bacterial genera presents in each group. **(B)** The relative abundance differs significantly among groups at the genus level. Different superscripts indicate significant differences according to the Kruskal-Wallis test (*P* < 0.05). Con, basal diet; G300, 600, 900, and 1200, basal diets complemented with 300, 600, 900, or 1200 mg/kg GML.

Taxonomic profiling indicated that the relative abundance of the phylum *Firmicutes* and *Bacteroidetes* altered obviously among the control and GML-treated groups (**Figure 2A**). The *Firmicutes/Bacteroidetes* ratio was calculated, which decreased in the ceca of 7-day-old broilers after 600 and 1200 mg/kg GML supplementation (*P* < 0.05) but not altered in each group on day 14 (**Figure 2B**). Supplementation with 600 and 1200 mg/kg GML increased the amount of *Bacteroidetes* relative to the amounts in the control, 300, and 900 mg/kg groups (*P* < 0.05; **Figure 2C**). The relative abundance of *Actinobacteria* decreased in the ceca of the 14-day-old broilers that received 600 and 900 mg/kg GML (*P* < 0.05; **Figure 2C**). Further, the relative abundance of the 20 predominant genera in each group was analyzed to illustrate the specific changes in the microbial taxa (**Figure 3A**). The results showed that the 300 mg/kg GML-treated group was enriched with *Barnesiella* and *CHKCI001* on days 7 and 14 (*P* < 0.05), respectively (**Figure 3B**). The enhanced proportion of *Coprobacter* and reduced *Lachnospiraceae_FE2018_group* were observed in the 600 mg/kg GML-treated group (*P* < 0.05). The relative abundances of *Barnesiella*, *Odoribacter*, and *Parabacteroides* increased (*P* < 0.05) in the broilers fed with 900 mg/kg GML (**Figure 3B**). In addition, except the decreased *Lachnospiraceae_FE2018_group*, 1200 mg/kg GML significantly enhanced the abundances of *Barnesiella*, *Faecalibacterium*, *Bacteroides*, *Odoribacter*, and *Parabacteroides* and *CHKC001* level in the ceca of the broilers (*P* < 0.05) relative to those in the control and broilers that received other GML dosages (**Figure 3B**).

**Figure 4:**
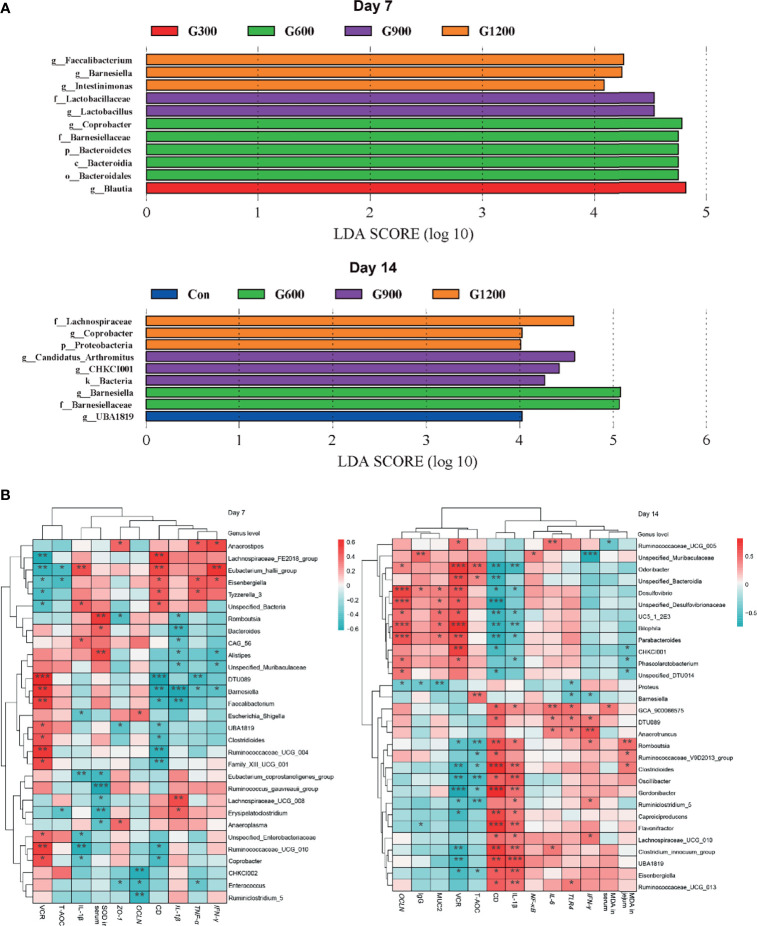
**(A)** LEfSe analysis of cecal microbiota (LDA score is greater than 4). **(B)** Heatmap of correlation among cecal microbiota, inflammatory factors, tight junctions, antioxidant enzymes, and intestinal integrity by Spearman analysis. *P < 0.05, **P < 0.01, ***P < 0.001. Con, basal diet; G300, 600, 900, and 1200, basal diets complemented with 300, 600, 900, or 1200 mg/kg GML.

The specific bacterial taxa associated with GML treatment was identified through linear discriminant analysis effect size (LEfSe, LDA score > 4) analysis. As shown in **Figure 4A**, only the genus *Blautia* was observed to be significantly abundant in the 300 mg/kg group. The 600 mg/kg GML-treated group showed the enrichment of the genus *Coprobacter*, family *Bacteroidetes*, phylum Bacteroidia, and order Bacteroidales. The family *Lactobacillaceae* and genus *Lactobacillus* predominated in the 900 mg/kg GML group. Furthermore, the genera *Faecalibacterium*, *Barnesiella*, and *Intestinimonas* were enriched in the ceca of 1200 mg/kg GML-treated broilers on day 7. At the 14th day of treatment, the abundance of genus *UBA1819* increased in the 300 mg/kg GML-treated group. LEFse analysis indicated significant distinctive bacteria of family *Barnesiellaceae* and genus *Barnesiella* in the 600 mg/kg GML-treated group. Genus *Arthromitus* and *CHKCI001* were overrepresented in the 900 mg/kg group. The cecal microbiota in the 1200 mg/kg GML-fed group was characterized by phylum, family *Lachnospiraceae*, and genus *Coprobacter*. Through Spearman correlation analysis, the relationships of changes in intestinal microflora at the genus level with intestinal integrity, inflammatory factors, antioxidant enzymes, tight junction proteins, and TLR/NF-κB signal pathway were discussed (**Figure 4B**). The levels of inflammatory factors were positively associated with the genera *Lachnospiraceae_UCG_008*, *Erysipelatoclostridium*, *Unspecified_Bacteria*, *Anaerostipes*, *Eubacterium_hallii_group*, *Eisenbergiell*a, *Lachnospiraceae_UCG_010*, and *Anaerotruncus* but negatively correlated with *Barnesiella*, *Faecalibacterium*, *Coprobacter*, *Odoribacter*, *Parabacteroides*, *CHKCI001*, and *Bilophila*. Intestinal integrity was possibly correlated with the genera *Coprobacter*, *Ruminococcaceae_UCG_010*, *Faecalibacterium*, *Barnesiella*, *Desulfovibrio*, *Bilophila*, *Parabacteroides*, *Phascolarctobacterium*, and *CHKCI001*. The abundances of *Lachnospiraceae_FE2018_group*, *Eubacterium_hallii_group*, *Ruminiclostridium_5*, *Enterococcus*, *Romboutsia*, and Caproiciproducens were negatively associated with intestinal barrier. The results indicated that the promoted intestinal health of broiler chicks may be relevant to the altered structure of the cecal microbiota manipulated by GML supplementation.

## Discussion

Medium-chain fatty acids (MCFA) have received widespread attention as feed additives, showing positive benefits and improving animal health, production, and feed digestibility (21, 22). Current evidence supports that MCFA and monoglycerides are generally effective in supporting growth performance and intestinal health (23). Feed intake is the basic feature that guides the growth rates of broilers (24). In the current study, dietary treatment with GML effectively increased FI rates of broiler chicks, consistent with previous findings (11). A combination of gut inflammation, immune cell infiltration, and increased levels of proinflammatory cytokines often reduce FI and cause diarrhea (25). Decreased levels of inflammatory factors after GML treatment may be one of the reasons for the increased FI in the present study. However, none of the supplemented treatment groups showed changes in BW, BWG, and FCR, consistent with the previous findings that GML did not alter the growth performance of broilers in the first 28 days of treatment (26). In addition, no effects were reported on the BW, BWG, and FCR after dietary supplementation with four GML levels (0, 1, 3, or 5 g/kg) during all experimental periods (3). Conversely, increased ADFI and BW and reduced FCR were observed in the 28–56-day-old yellow-feathered broilers with dietary GML (16). Broilers fed with GML had increased BWG, ADG, and feed consumption and decreased FCR (11). The positive effect of MCFA on digestibility and growth performance is the inhibition of pathogen proliferation (27).

Diets complemented with the glycerol-esters of MCFAs exert immunomodulatory effects (28). In the current study, reduced IL-1β and TNF-α and increased IgG levels were observed in the sera after dietary treatment with GML. Proinflammatory cytokines IL-6 and TNF-β levels decreased in the sera after GML supplementation, which alleviates systemic inflammatory response in high-fat-diet-fed mice (6). Moreover, GML may be considered a topical anti-inflammatory agent (7). It down-regulated the gene expression of jejunal *IL-1β*, *IL-6*, and *IFN-γ* in the present study. Owing to the relatively stable state and long residence time in the gastrointestinal tract, an anti-inflammatory environment was induced instead of systemic inflammation after the administration of a high dosage of GML (15). A similar result was observed in this study. The 1200 mg/kg GML dosage showed better effects on broiler inflammation and immunity. Monoglycerides and MCFAs exhibit antimicrobial and immunomodulatory activities as additive candidates, thereby mitigating feed pathogen proliferation and improving enteric health in weaned pigs (23). An *in vitro* study showed that lauric acid treatment reduces the concentration of proinflammatory cytokines (TNF-α, IL-6, and IL-1β) in the culture supernatant of microglia attacked by LPS (29). Moreover, GML disrupted the lipid dynamics of human T cells, potentially reducing the TCR-induced production of cytokines (5). This function suggests that GML has an immunomodulatory role.

One of the central ways feed compounds affect immunity is activating NF-κB, which is an inducible central regulator of inflammatory responses involved in most innate immune receptor signaling pathways (30). In this study, GML supplementation decreased jejunal *NF-κB* expression. Lauric acid and GML have minimal impacts on NF-κB activation in the absence of an LPS challenge, although a statistically significant increase can be observed at certain concentrations (30). Such increase partially explains the results of the present study. The canonical pathway of NF-κB activation involves signaling by pattern recognition receptors, such as TLR (31). The data indicated that dietary treatment with 600 and 1200 mg/kg GML effectively reduced the relative mRNA expression of *TLR4*. Similar results were obtained in a mice experiment in which GML down-regulated *TLR2* and *MyD88* expression in the liver, reducing systemic inflammation in high-fat-diet fed mice (6). Lauric acid increases the activity of NF-κB through the dimerization of TLR2 and TLR1 or TLR6 and the activation of TLR4 (32).

Intestinal integrity is a key factor for preventing the invasion of pathogenic microorganisms in broilers (33). Dietary GML tends to increase the VH and VCR and decrease the CD of the jejunum, thus improving growth performance (16). In this study, dietary GML decreased CD and increased VCR in the jejuna of broilers with increasing dosages. High VCR is widely regarded as a good indicator of mucosal turnover and is related to strong digestion and absorption capacity (33). Natural extracts with anti-inflammatory effects restore the damaged intestinal morphology of broilers attacked by LPS (34). Therefore, the improvement in intestinal integrity can be partially explained by the decreased expression of proinflammatory cytokines and down-regulation of *TLR4/NF-κB* signal transduction. After GML was given to the mice, the normal expression levels of *ZO-1*, *occludin*, *claudin-1*, *jam-1*, and *MUC 2* suggested that GML maintained the mucosal barrier and intestinal health (15). In this study, dietary GML effectively benefited the jejunal MUC 2 content and up-regulated *ZO-1* and *occludin* expression, demonstrating the beneficial effect of GML on intestinal barrier of broilers. The TNF-α and IFN-γ are related to the reduction in epithelial barrier function and increases the permeability of the mucosal barrier (35). The reduction in serum TNF-α level and decrease in jejunal *IFN-γ* expression in this study supported the idea that GML mediates intestinal barrier function through a mechanism associated with the attenuation of intestinal inflammation.

Oxidative stress is an important factor for the destruction of mucosal barrier function (36). In the current study, reduced MDA content and increased T-SOD and T-AOC activity in the sera and jejuna indicated that GML reduced lipid peroxidation and improved the antioxidant capacity of the broilers. This result is in a line with a previous study (12). In laying hens, increase in SOD level and reduced glutathione and MDA levels were observed, suggesting that GML can decrease lipid peroxidation and enhance the antioxidant capacities of broilers. These features are beneficial to growth performance (26). The peroxidation level in the meat of GML-fed chickens was reduced and proportional to the increase in dietary additive concentration (11). Significant connections between inflammation and oxidative stress have been found. These processes induce each other reciprocally, thereby establishing a vicious cycle that perpetuates and propagates inflammatory response (20). Moreover, the proinflammatory signaling cascades triggered by TLR engagement enhance the expression of iNOS, implying that TLR activation may result in oxidative stress (37). Thus, the improvement in antioxidant capacity in the GML-treated broilers may be related to lowered inflammatory response and down-regulated TLR4/NF-κB pathway. Reduced oxidative stress was beneficial for relieving inflammation.

Intestinal microbiota contributes to the maintenance of intestinal physiological structure and function, which are considered relevant to intestinal inflammation, barrier function, and growth performance of a host (38). In the present study, dietary GML affected the cecal microbial composition, with altered alpha- and beta-diversity indices. The results of alpha diversity were different from the previous study that GML did not show any effect on alpha diversity of yellow-feathered broilers (16). In mice, 1600 mg/kg GML led to a decrease in Shannon and Simpson indices but increased microbial diversity in high fat diet-fed mice (6, 15). Modulatory effects of GML on beta-diversity were consistent from different studies (6, 15, 16), which indicated that dietary GML modulated the microbial composition of the host. A rich community of species enhances the stability of the intestinal microecology and may be related to reduced sensitivity to bacterial invasion and intestinal inflammation (39). Thus, the alleviated intestinal inflammation of broilers may be associated with the modulated structure and increased diversity of intestinal microbial community structure after GML addition. At the phylum level, GML mainly altered the relative frequency of *Bacteroidetes* and *Firmicutes* in the ceca of the broilers, which was consistent with previous findings (16). Increase in *Actinobacteria* and *Firmicutes*/*Bacteroidetes* ratio is the pattern of an impaired intestinal barrier (40). In this study, the cecal microbiota in GML-treated broilers was characterized by decreased level of *Actinobacteria* and *Firmicutes*/*Bacteroidetes* ratio, which may be the potential reasons for the improved intestinal barrier. In addition, 1200 mg/kg GML increased the amount of cecal *Bacteroidetes*, which mainly contributes to the fermentation of indigestible carbohydrates to butyrate and exerts beneficial effects on mucosal barrier integrity through its anti-inflammatory effects (41, 42). Dietary GML can promote colonization of some certainly beneficial bacteria (16). Mice fed with 400 and 800 mg/kg GML had higher abundances of *Barnesiella*, which was considered to be positively associated with a healthy state (15). In yellow-feathered broilers, *Lachnospiraceae* were observed with increased colonization by GML supplementation (16), which can promote health by producing host nutrients and providing an energy supply to the colonic epithelium, as well as maintaining host immune homeostasis (43). *Parabacteroides* are associated with T-cell differentiation by enhancing and maintaining the IL-10-producing Treg cells (44). In the present study, the relative abundance of *Barnesiella*, *Coprobacter*, *Lachnospiraceae*, *Faecalibacterium*, *Bacteroides*, *Odoriacter*, *Parabacteroides*, and *CHKCI001* were higher in the GML-treated groups, especially under the dosage of 1200 mg/kg. In line with previous findings (15, 16), dietary GML improved intestinal health by promoting the colonization of beneficial bacteria. In addition, *Barnesiella*, *Faecalibacterium*, *Bacteroides*, *Odoribacter*, and *Parabacteroides* were positively correlated with the level of SCFAs and produced butyrate, which exerted immunomodulatory and anti-inflammatory effects by mediating the homeostasis of colonic regulatory T cell populations and promoted intestinal integrity (45). In the current study, the promoted intestinal morphology by dietary GML may be related to increased SCFAs levels, which was consistent with results observed in yellow-feathered broilers and mice (15, 16). Butyrate with antioxidant properties modulates inflammatory response by inhibiting NF-κB and provides energy to the intestinal epithelial cells (40). A significant impact was reported on host health and physiology after GML supplementation, which directly acts on the intestinal microbiota and considerably affects metabolism and immunity (46). Therefore, the increased abundance of butyrate-producing bacteria after GML addition was associated with alleviated intestinal inflammation and improved intestinal morphology of the broilers.

In conclusion, dietary GML ameliorated intestinal morphology and barrier function of broiler chicks by ameliorating inflammation and promoting antioxidant status. Our results confirmed the immunomodulatory, antioxidant, and anti-inflammatory properties of GML, which may be associated with the suppression of the TLR4/NF-κB signaling pathway. The altered structure of the cecal microbiota manipulated by GML may be the main reason for the promotion of intestinal health in the broilers.

## Data Availability Statement

The original contributions presented in the study are publicly available. This data can be found here: https://www.ncbi.nlm.nih.gov/bioproject/732475.

## Ethics Statement

The animal study was reviewed and approved by Ethics Committee of the Shandong Agricultural University.

## Author Contributions

ZS conceived the idea and provided resources. LK designed the study, performed the experiment, and wrote the manuscript. ZW and ZS provided guidelines. CX and QZ participated in the experiment. All authors contributed to the article and approved the submitted version.

## Funding

This work was supported by the Natural Science Foundation of Shandong Province (ZR2020MC170), the National Key R&D Program of China (2018YFD0501401-3), and the Shandong Province Agricultural Industry Technology (SDAIT-11-08).

## Conflict of Interest

The authors declare that the research was conducted in the absence of any commercial or financial relationships that could be construed as a potential conflict of interest.

## Publisher’s Note

All claims expressed in this article are solely those of the authors and do not necessarily represent those of their affiliated organizations, or those of the publisher, the editors and the reviewers. Any product that may be evaluated in this article, or claim that may be made by its manufacturer, is not guaranteed or endorsed by the publisher.
